# The challenges of viral hepatitis elimination: a global response to a global problem

**DOI:** 10.1186/s12889-023-15960-w

**Published:** 2023-06-01

**Authors:** Antony P. Black, Jack Wallace, Mawuena Binka, Zahid Ahmad Butt

**Affiliations:** 1 Laboratory for Vaccine-Preventable Diseases, Institut Pasteur du Laos, Vientiane, Laos; 2 Burnet Institute, Melbourne, Australia; 3 Centre for Social Research in Health, UNSW, Kensington, Australia; 4 School of Population and Public Health, University of British Columbia, Vancouver, Canada; 5 School of Public Health Sciences, University of Waterloo, Waterloo, Canada

## Main text

Viral hepatitis is responsible for millions of chronic infections and deaths worldwide [1]. Often asymptomatic in the acute phase of illness, hepatitis C (HCV) and hepatitis B (HBV) viruses were responsible for 96% of viral hepatitis associated deaths in 2015, and remain major sources of morbidity and mortality globally [2]. Hepatitis D virus (HDV) infection exacerbates the outcome of HBV infection, while hepatitis A (HAV) and hepatitis E (HEV) viruses mostly cause acute infections. Together the threat they pose to public health prompted the World Health Organization (WHO) to establish a viral elimination agenda [3].

In 2020, the Nobel Prize for Medicine or Physiology was awarded to Drs. Harvey Alter, Michael Houghton and Charles Rice for their role in the discovery and characterisation of HCV, which is mainly transmitted through percutaneous routes by reusing non-sterile medical equipment or sharing drug injecting equipment, or unsafe transfusions [4]. The decades following their seminal work have seen the development of sensitive serological and nucleic acid tests for the virus, and, more recently, the advent of direct-acting antivirals (DAA). The transformation of a chronic, debilitating, and often fatal disease into a curable infection has contributed to the goal of the WHO to more than halve the number of current annual HCV infections by 2030 [1]. Nevertheless, significant obstacles remain and over 50 million individuals are estimated to be chronically infected with HCV worldwide, [5] many without access to this life saving cure. In some settings, HCV infection is most common among marginalised populations such as people who inject drugs who often suffer stigma and, consequently, are reluctant to be tested for infection or unwilling to access health care services. Furthermore, despite improved testing protocols, such as prequalified rapid tests and simplified nucleic acid screening, and a reduction in cost of DAA in some settings, difficulty in accessing testing and treatment by at-risk populations remains a major issue. Suggested solutions to these barriers to care include decentralised, integrated care and access to generic DAAs, which may address these issues to some extent but need to be implemented at national and global levels to be effective [6]. Another important aspect in achieving HCV elimination goals is to document the HCV cascade of care, from diagnosis and linkage with care to treatment and cure, to support evaluating access and service delivery as well as effectiveness of HCV elimination programmes. Concurrently, epidemiological studies of HCV, including modelling studies, are crucial to predict and demonstrate the impact of testing and treatment strategies, as well as highlighting the obstacles needing to be overcome to ensure equity of access to testing and treatment.

In contrast to HCV, chronic infection with HBV is presently not curable and requires long-term monitoring and treatment. The WHO estimated that approximately 296 million people worldwide were chronically infected in 2019, with a consequent greater risk of death from cirrhosis and liver cancer [7]. Many infections continue to occur in Africa and Asia where the main routes of transmission are horizontal and perinatal, respectively. The WHO viral hepatitis elimination plan includes a target to reduce new HBV infections from 1.5 million in 2020 to 170,000 in 2030 [1]. For individuals with chronic infection, testing, long-term monitoring and treatment remain a challenge in resource-limited settings due to the high costs and lack of human resources. However, safe and effective HBV vaccines exist, and are administered routinely in many endemic countries at birth, during infancy, and to particular adult risk groups such as health care workers. The impact of the HBV vaccine is well documented in several settings, with a great reduction in virus exposure as well as chronic infection and subsequent liver disease [8]. Low and inequitable vaccine coverage impedes this reduction in prevalence in several locations where HBV remains endemic. Missing timely HBV birth doses, crucial for preventing early vertical transmission, can be a particular issue in areas where unattended home-births and/or vaccine stock-outs are common. Similarly, unclear national guidelines can perpetuate a lack of occupational health policy, for example, regarding vaccination of healthcare workers and blood donor screening. Epidemiological studies are crucial to inform policy and to demonstrate impact of different vaccine coverage and testing and treatment strategies on exposure and chronic infection and rates of liver disease. HDV is an incomplete virus that needs HBV to replicate and cause infection and can cause severe liver damage and increase the risk of liver failure and liver cancer. Thus, HDV can be prevented through vaccination against HBV.

Unlike HCV and HBV, HAV and HEV usually do not form chronic infection and are not associated with liver cancer. Nevertheless, they can cause severe disease and death, for example HEV infection during late pregnancy poses a high risk of acute liver failure, foetal loss or death. The epidemiology of both infections is important and varies greatly by location, due to the different vaccination policies and risk practices for example, inequitable access to good sanitation (risk factor for both) and contact with swine (a source of HEV genotype 3 and 4). In many locations, sanitation improvements and reduction of other common risks have resulted in lower seroprevalence of protective antibodies and therefore high number of susceptible individuals and occurrence of large outbreaks [9, 10].

The changing epidemiology of HAV, HBV, HCV, HDV and HEV is fascinating and context-specific with social and health service delivery implications. We are pleased to announce the launch of this BMC Public Health collection and welcome submissions from a broad range of academic disciplines that increase our understanding of, and responses to, the implications of the changing epidemiology of viral hepatitis. We encourage original research articles covering any aspect of this topic, such as public health interventions, surveillance data, and others. The target audience includes academic researchers, educators, clinicians and policy makers, with an aim to provide a platform for freely available, up-to-date information in this rapidly progressing field.

## Data Availability

Not applicable.
