# Correction: The challenges of viral hepatitis elimination: a global response to a global problem

**DOI:** 10.1186/s12889-023-16096-7

**Published:** 2023-06-28

**Authors:** Antony P. Black, Jack Wallace, Mawuena Binka, Zahid Ahmad Butt

**Affiliations:** 1 Laboratory for Vaccine-Preventable Diseases, Institut Pasteur du Laos, Vientiane, Laos; 2 Burnet Institute, Melbourne, Australia; 3 Centre for Social Research in Health, UNSW, Kensington, Australia; 4 School of Population and Public Health, University of British Columbia, Vancouver, Canada; 5 School of Public Health Sciences, University of Waterloo, Waterloo, Canada

**BMC Public Health (2023) 23:1042**

10.1186/s12889-023-15960-w

In the original publication of this article the corresponding authorship of Zahid Ahmad Butt was not indicated. The original publication has been updated to amend this.

